# Gender stereotypes and social perception of vocal confidence is mitigated by salience of socio-indexical cues to gender

**DOI:** 10.3389/fpsyg.2023.1125164

**Published:** 2023-12-14

**Authors:** Jennifer M. Roche, Katie Asaro, Bradley J. Morris, Shae D. Morgan

**Affiliations:** ^1^Educational Psychology, Kent State University, Kent, OH, United States; ^2^Speech Pathology & Audiology, Kent State University, Kent, OH, United States; ^3^Otolaryngology, Head and Neck Surgery and Communicative Disorders, University of Louisville, Louisville, KY, United States

**Keywords:** vocal confidence, socio-indexical cues, heuristics, stereotypes, gender

## Abstract

**Introduction:**

Socio-indexical cues to gender and vocal affect often interact and sometimes lead listeners to make differential judgements of affective intent based on the gender of the speaker. Previous research suggests that rising intonation is a common cue that both women and men produce to communicate lack of confidence, but listeners are more sensitive to this cue when it is produced by women. Some speech perception theories assume that listeners will track conditional statistics of speech and language cues (e.g., frequency of the socio-indexical cues to gender and affect) in their listening and communication environments during speech perception. It is currently less clear if these conditional statistics will impact listener ratings when context varies (e.g., number of talkers).

**Methods:**

To test this, we presented listeners with vocal utterances from one female and one male-pitched voice (single talker condition) or many female/male-pitched voices (4 female voices; 4 female voices pitch-shifted to a male range) to examine how they impacted perceptions of talker confidence.

**Results:**

Results indicated that when one voice was evaluated, listeners defaulted to the gender stereotype that the female voice using rising intonation (a cue to lack of confidence) was less confident than the male-pitched voice (using the same cue). However, in the multi-talker condition, this effect went away and listeners equally rated the confidence of the female and male-pitched voices.

**Discussion:**

Findings support dual process theories of information processing, such that listeners may rely on heuristics when speech perception is devoid of context, but when there are no differentiating qualities across talkers (regardless of gender), listeners may be ideal adapters who focus on only the relevant cues.

## 1 Introduction

In contemporary US culture, many recognize that gender does not exist along a gender binary. Yet, studies in social cognition still find that social judgments are often based on gender (binary) stereotypes (Fisk and Ridgeway, 2018). This has major implications for women in professional settings, as gender stereotypes may impact equitability. For instance, Prentice and Carranza (2002) found that gender prescriptions (what women and men should be) are more highly associated with men being confident (e.g., leadership ability, ambition, higher self-esteem, assertiveness, and decisiveness) and women being passive and agreeable. Men are often rated as more confident and agentic than women (Fisk and Ridgeway, 2018), but when women exhibit assertiveness traits, they are often rated more severely and suffer social and professional backlash (e.g., overlooked for professional advancement; Amanatullah and Tinsley, 2013). Perceptions of confidence are important because they allow us to command respect from others, influence our social status, persuade others, and communicate trust through knowledge and certainty (Heesacker et al., 1983; Booth-Butterfield and Gutowski, 1993; Driskell et al., 1993; Carli et al., 1995; Jiang and Pell, 2015, 2016, 2017; Mori and Pell, 2019)—which may be important social goals to women and men alike. To close this assumed gender *communication* gap, it is important to consider how adapting the interpretation of socio-indexical cues (i.e., social cues that relate to the context; Clark, 1998; Pajak et al., 2016; Yu, 2022—e.g., social features of who is saying it may shape interpretation; Babel and Russell, 2015) away from common gender stereotypes could positively impact women in society.

Rising intonation, for example, is a cue that women and men may use to communicate a number of things in different contexts (Warren, 2016). Perhaps the most considered is the use of rising intonation to indicate a question (e.g., queclarative; Sadock, 1974; Geluykens, 1987; Brown et al., 2015; Warren, 2016). However, we may also use this cue to communicate pragmatic information in social contexts. For example, *uptalk* (e.g., *valley girl talk*), which is also marked by a rising intonation or pitch contour, is commonly used as a performative act to communicate deference or insecurity (Warren, 2016). Warren and Britain (2000) suggested that rising intonation may also be used as a way to back-channel or a way to check in with a communication partner to assess understanding. We may also use rising intonation to communicate uncertainty (Ward and Hirschberg, 1985) and/or a lack of confidence (Roche et al., 2019, 2022). Roche et al. (2022) found that both women and men produce rising intonation to convey a lack of confidence and declining intonation to convey increased confidence. However, women talkers tended to be perceived as less confident overall, especially when they used the rising intonation contour. This may be related to how the women talkers used rising intonation, or it may have been related to how socio-indexical cues to gender and effect interact to shape social judgments about speaker intention.

We, therefore, should first consider the role of socio-indexical cues on cognition during social judgments. Cognition is heavily involved in the evaluation of socio-indexical cues that are used to form exemplars and social schemas that promote generalizations and result in easier parsing of communication (Ladefoged and Broadbent, 1957; Babel and Russell, 2015). Social indices often help us interpret pragmatic communication—some types of indices include information about a talker's gender (Strand, 1999), age (Drager, 2011), cultural origin (Clopper and Pisoni, 2004), sexual orientation (Munson et al., 2006), communication difficulties (e.g., fluency disorders; Klouda and Cooper, 1988; Roche et al., 2021), and even affective states (Nygaard and Queen, 2008; Morgan, 2019). Dual process models of persuasion (i.e., an information processing model that assumes persuasion happens in one of two information processing modes—heuristic vs. systematic; Chaiken, 1980) account for the cognitive processes associated with aiding the interpretation of intent through consideration of socio-indexical cues.

In fact, listeners may use heuristic (e.g., cognitive shortcuts; Tversky and Kahneman, 1974) and/or systematic (deep and effortful) based processes to attend to important *vocal* cues to interpret meaning (e.g., Elaboration Likelihood Model; Chaiken, 1980; Petty and Cacioppo, 1986; Guyer et al., 2021). The development of social generalizations from heuristics derived from commonly occurring schemas of socio-indexical cues may aid listeners in making social judgments (Clark, 1998; Babel and McGuire, 2015; Roche et al., 2020). These generalizations typically come from the interpretation of these cues and the regularity with which they occur within the person's social context (Linville and Jones, 1980; Linville, 1982) to form exemplars that may be more easily accessed later (Goldinger, 1996, 1998; Johnson and Mullennix, 1997). These generalized exemplars and schemas typically help us more easily understand our social world by creating cognitive dimensions used to characterize, judge, and compare others (Jussim et al., 1987). We, therefore, develop *assumed characteristics* (e.g., of the speaker; Locksley et al., 1982; Jussim et al., 1987) to help us understand the important and relevant information about our conversation partners. Unfortunately, these generalized schemas sometimes result in harmful stereotypes (Greenwald and Banaji, 1995), but it is less clear how different social indices produced through the vocal channel may interact to guide interpretation in favor of heuristic over systematic processing.

Heuristic-based processes may sometimes be driven by stereotypes, while systematic-based processing is likely to rely on deeper interpretation of so-called persuasive variables that listeners will use to interpret meaning (McGuire, 1969; e.g., channel, message, receiver, and destination; cited in London et al., 1971, p. 359). Therefore, socio-indexical cues may trigger (e.g., Heuristic Information Processing; Chaiken, 1980) which processing path is selected—heuristic, based on a stereotype—or systematic—requiring deeper processing. When considering social indices for the interpretation of prescriptive communicative behaviors among women and men (e.g., when communicating confidently), we may better understand how socio-indexical cues affect gender interactions to guide perception.

In addition to these socio-indexical cues, the meaning of the words that speakers choose to use when communicating may also impact judgments of stereotypes (Beukeboom and Burgers, 2019). Mulac et al. (2000) found that men tended to use more negations than women. While negation is much harder on the cognitive system to process (relative to affirmation; Wason and Jones, 1963; Clark and Chase, 1972; Just and Carpenter, 1976), Dale and Duran (2011) argue that contextual factors crucially impact how cognitive processing handles negation. Using words related to negation and affirmation likely impacts the perception of agreeableness among women and men. For example, when women produce a brief pause before an affirmation, they are perceived as less agreeable than men (Roberts and Francis, 2013; Kendrick and Torreira, 2015; Roberts and Norris, 2016). However, when no pause is presented, women and men tend to be rated equitably on agreeableness (Roberts and Norris, 2016). Winter et al. (2021) found that cognitive flexibility (i.e., consideration of broader representations beyond a dominant one, Winter et al., 2021, p. 8) was enhanced when negations were used in communication. Their study found evidence that the inclusion of negations was effective in improving outgroup trust and cognitive flexibility in listeners. Therefore, it is important to consider language use in the context of interpreting socio-indexical cues to inform our understanding of how stereotypes impact social judgments about others.

Moreover, theories about speech perception assume that listeners will take advantage of statistical contingencies associated with language and socio-indexical cues (Nygaard et al., 1994; Johnson et al., 1999; Strand, 1999; Mitchel et al., 2016; Kleinschmidt, 2019) and listeners will draw on expectations about cue-mapping based on socio-indexical group membership. Taken together with dual process models of information processing, triggering cues and statistical contingencies in the communicative environment may contribute to processing differences and reliance on or rejection of harmful stereotypes. Kleinschmidt (2019) argues that ideal adapters may make predictions that help them track conditional distributions among groups that are informative for speech recognition. Therefore, when no other information or context is provided, listeners may default to gender stereotypes that are informed by heuristics to make easier social-categorical judgments. For example, if someone holds the cognitive representation that women are less confident than men and when they hear a cue suggesting a lack of confidence (e.g., rising intonation), they may be more likely to interpret it consistent with their own bias (Beukeboom et al., 2010; Roche et al., 2019, 2020). However, listeners may also be able to learn specific distributional statistics when encountering cues that do not vary between genders. In fact, there is likely no benefit to tracking separate cue distributions for groups that do not systematically vary (Kleinschmidt, 2019). Therefore, when many women and men produce similar cues indicating confidence and words of affirmation and negation, listeners may be less likely to differentiate between genders when interpreting the affective intent of the language used.

This led to the following research question: How does context drive social judgments of confidence? First, it was hypothesized that a female speaker would be judged less confidently than a male-pitched speaker (consistent with Roche et al., 2019, 2022) because confidence is often viewed as a male-typed cue (Fisk and Ridgeway, 2018). However, when many women and many men produce similar language and vocal cues suggesting a lack of confidence, then listeners may equally judge women's and men's confidence because there is no cognitive benefit to tracking separate cue distributions between men and women (Kleinschmidt, 2019). Language cues may also impact perceptions of confidence, such that negation would likely recruit higher ratings of confidence because affirmations are more likely to be a *woman-typed* cue that is associated with agreeableness (i.e., Roberts and Norris, 2016) and because women tend to be rated as less confident than men (Fisk and Ridgeway, 2018; Roche et al., 2022). To test this, we evaluated the interaction between language (i.e., affirmation and negation) and socio-indexical cues to affective expressions of confidence and speaker gender cues in single and multi-talker listening conditions in a social judgment task.

## 2 Method

### 2.1 Participants

Participants included a total of 207 undergraduate students (single talker: *n* = 92; multiple talkers mixed: *n* = 59; multiple talkers blocked: *n* = 56) recruited from an upper Midwestern University (women = 178, men = 29; mean_age_ = 20.78; SD_age_ = 3.9 years). Seven participants were excluded for reporting not using headphones (*N* = 200; excluded: single *n* = 1; multiple talkers mixed *n* = 1; multiple talkers blocked *n* = 5). All participants were native speakers of American English with no self-reported diagnosis of any speech, language, or hearing impairment with normal to corrected-normal vision. A larger number of participants were recruited for the single talker task because there were fewer experimental trials than in the multiple talker tasks.

### 2.2 Platform

FindingFive (FindingFive Team, 2019), an online, cloud-based platform designed by cognitive scientists for psychological and behavioral research studies, was used to collect participant data. Participants joined the study using their own equipment—a laptop or desktop computer and headphones were a requisite for study completion. Any participant not using headphones was excluded. Additionally, question stimuli were selected from the Mindpixel Corpus (McKinstry et al., 2008), which is a web-based collaborative artificial intelligence project that aimed to collect validated true and false statements from millions of humans (between 2000 and 2005). Praat (Boersma and Weenink, 2023) and Audacity were used to implement the acoustic manipulations.

### 2.3 Stimuli

The stimuli included three trivia questions selected from the Mindpixel corpus that ranged in veridicality (not true: 0%; ambiguous: 50% true; completely true: 100%; McKinstry et al., 2008; see Table 1 for the questions). Confidence is expressed under a range of truths; therefore, a range of question truth values was selected to increase the ecological validity and mundane realism of the task.

**Table 1:** Questions used in the current study were taken from the Mindpixel corpus with veridicality values of 0% true, 50% true, and 100% true.

**Truth value**	**Question**
0%	Are cats and dogs the same animal?
50%	Can bacteria live in boiling water?
100%	Is a leopard a cat?

#### 2.3.1 Vocal stimuli

Four cis-gender women were recruited to produce a vocal recording of the words *yeah* and *no*, in a neutral tone of voice [i.e., all women produced a fundamental frequency (*f*_0_) within a typical range for women; *f*_0_ range: 182.98–207.91 Hz, mean *f*_0_ = 188.77, SD = 17.09]. As a note, many of the women naturally produced a slight rising intonation in their natural productions, but efforts were taken to reduce the natural rising intonation by flattening the intonation contour by the talkers *f*_0_ in Praat. These natural productions acted as filler trials in our matched guise technique (Ball and Giles, 1982). Critical trials included modification of the pitch contour of each woman's natural productions with a rising or declining intonation contour. After the vocal confidence pitch manipulation was implemented, we modified the perceived gender of the women's natural utterances—participants accurately identified the women speakers as women ~98% of the time. As a note, only female-pitched voices were used because the attempt at using a base male voice produced a gender category that sounded ambiguous based on a gender binary, whereas the female voices produced a male-pitched voice that more closely approximated a biological male.

##### 2.3.1.1 Acoustic manipulation

*Yeah* was chosen over *yes* to make it simpler to implement the acoustic modifications along the vowel portion of the word that occurred at the end of the utterance. Prior to the critical study manipulations (i.e., gender and intonation), we equated the pitch contour shape, amplitude, and speaking duration across all talkers. Some talkers naturally produced uptalk variation, and to create the neutral or flat intonation stimuli, each talker's natural pitch contour was removed and replaced with a flat contour based on their *f*_0_. We also equated the root-mean-square amplitude to 62.7 dB and the length of all recordings to meet a 500 ms cutoff, resulting in shortening and lengthening of the vowel portion of each utterance where necessary.

Once these initial edits to the original sound files were completed, we then adjusted the intonation contour for each talker's productions to include additional categories of confident (declining intonation) or not confident (rising intonation) (based on Roche et al., 2022) using Praat. To create these pitch contours, the talkers' flat/neutral pitch contour (i.e., based on their *f*_0_) was used halved (+/– the *f*_0_) at the last pitch point in the natural utterance. This produced the perception of rising intonation (i.e., not confident) and declining intonation (i.e., confident), consistent with Roche et al. (2019, 2022).

After manipulating the intonation, each sound file was opened and further manipulated in Audacity to change the perceived gender of the speaker from a female voice to a male-pitched voice. Though pitch is not the only acoustic cue that differentiates gender (e.g., formant frequencies are also important cues to gender; Gelfer and Mikos, 2005; Gelfer and Bennett, 2013; Weirich and Simpson, 2018), we only chose to manipulate pitch in the current study, because we were specifically interested in how pitch affected the expression of confidence. While this provided more control in the experiment, the male-pitched speakers did not truly reflect a biological male speaker's vocal quality. Nevertheless, it did provide an approximation and a vocal category that was qualitatively different than the woman speakers' voices (male-pitched range = 144.38–168.38 Hz, mean = 152.85 Hz, SD = 13.85 Hz). In fact, at the end of the experiment, when participants were asked to identify the gender of the speaker, they were significantly more accurate at identifying the women speakers as women (mean = 0.98, SD = 0.13) relative to the male-pitched speakers as men (*t* = 9.92, *p* < 0.001), but the identification of the male-pitched speakers as men was still above chance (mean = 0.89; SD = 0.31). This suggests that the male-pitch manipulation was effective.

To change perceived gender, we used the ‘Change Pitch' procedure under Effect and set the percent change to −19, which produced a vocal quality that approximated a biological male's pitch. This resulted in four additional voice stimuli, summing to eight (four female voices and four male-pitched voices) total. We chose to manipulate the pitch contour before the gender manipulation to make the pitch contour magnitude consistent when the gender manipulation was implemented.

**Figure 1:**
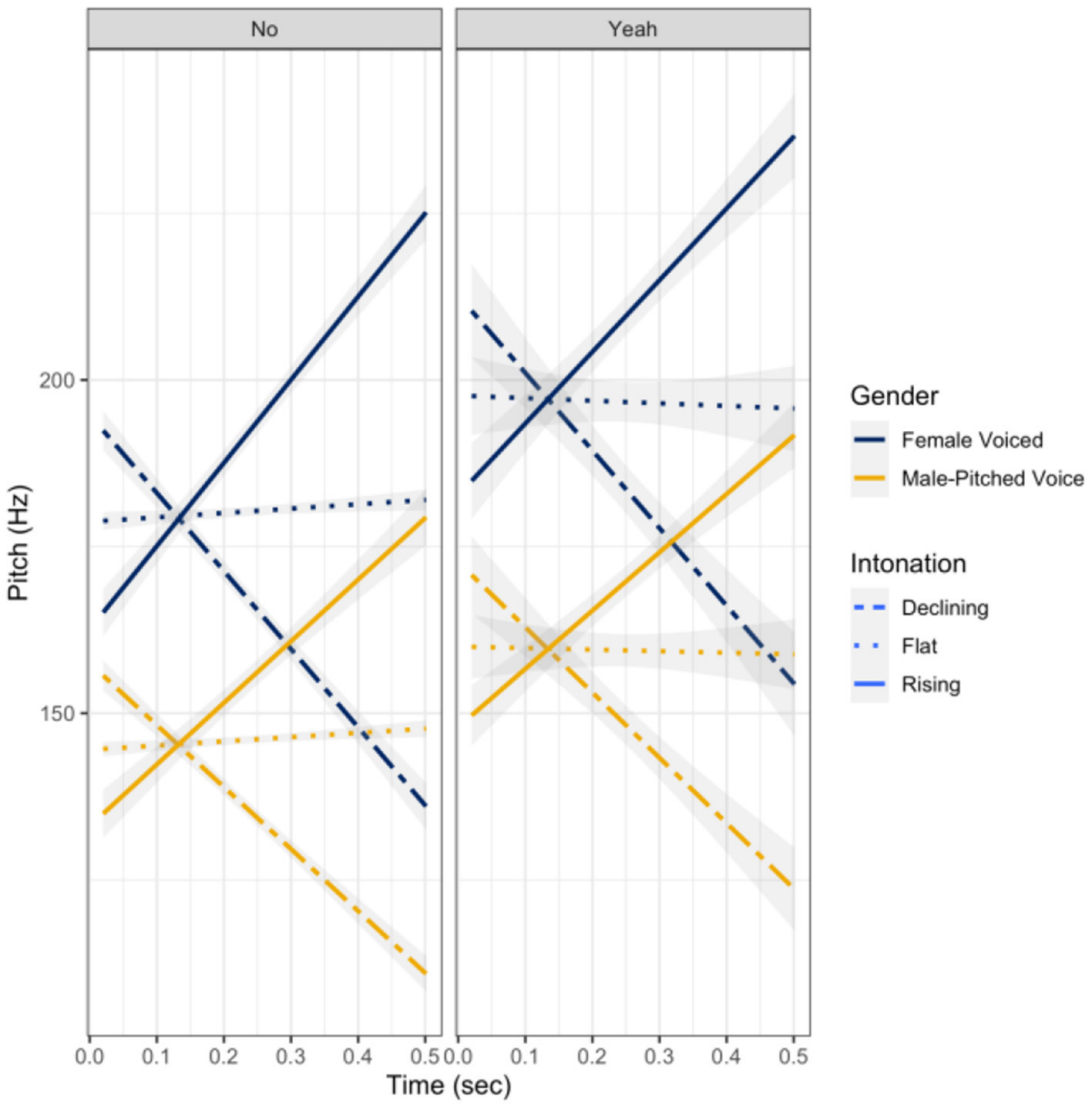
Pitch contour shape (*f*_0_ in Hz by time in seconds) post intonation and gender pitch manipulation.

To ensure that the pitch contour and gender manipulations were relatively similar across talkers, the magnitude of the pitch slopes of each of the sound files was evaluated using repeated measures ANOVA.^1^ The repeated measures ANOVA indicated there were significant differences in the magnitude of the slopes between the different intonation patterns (declining, flat, rising): *F*_(2, 33)_ = 179.88, *p* < 0.001; however, talker gender, word, or the interactions produced any slope differences (all *p* > 0.05). As can be seen in Figure 1, *yeah* utterances were slightly elevated in pitch relative to the *no* utterances, but the pitch slopes were not significantly different between words: *F*_(1, 33)_ = 0.43, *p* = 0.52. The declining and rising intonations were relatively uniform in degree across talkers; some talker variability did exist, but not significantly: *F*_(2, 33)_ = 2.95, *p* = 0.07. Moreover, there were slight (visually) natural rising intonations with the *no* utterances and slight natural declinations for the *yeah* utterances, but these contours were still relatively flat as compared to the manipulated rising and declining intonations—though not significant: *F*_(2, 33)_ = 0.281, *p* = 0.76.

## 3 Design and procedure

A two-voice (female; male-pitched) three-intonation (natural, rising intonation, and declining intonation) design was used for each subject under three different experimental conditions (single talker; multiple talkers mixed; and multiple talkers blocked). In the single talker condition (see Figure 1 for an example stimulus screen), listeners were presented with sound files from one female voice and one male-pitched voice (i.e., the male-pitched voice was derived from the woman talker's *yeah* and *no* responses). In the multi-talker mixed condition, listeners randomly heard responses paired with the three questions from all four female and four male-pitched voices. Finally, listeners in the multi-talker blocked condition either heard only the female voice utterances paired with the questions first followed by the male-pitched utterances paired with the questions (female voice 1st condition) or they first heard the male-pitched voice utterances alone paired with the questions first (male-pitched voice 1st condition) followed by the female voices paired with the questions.

Additionally, listeners were presented with the *yeah* and *no* files manipulated by the intonational contour (flat intonation, rising intonation, and declining intonation). Based on results from other studies, it was assumed that sound files with rising intonation would be perceived as less confident, while sound files with declining intonation would be perceived as more confident (Jiang and Pell, 2015; Roche et al., 2019, 2022). We had no a priori assumptions about the natural/flat productions and included them mainly to increase the number of trials so that listeners would not guess the purpose of the task (i.e., consistent with the matched guise technique, Ball and Giles, 1982), and these trials acted as fillers.

**Figure 2:**
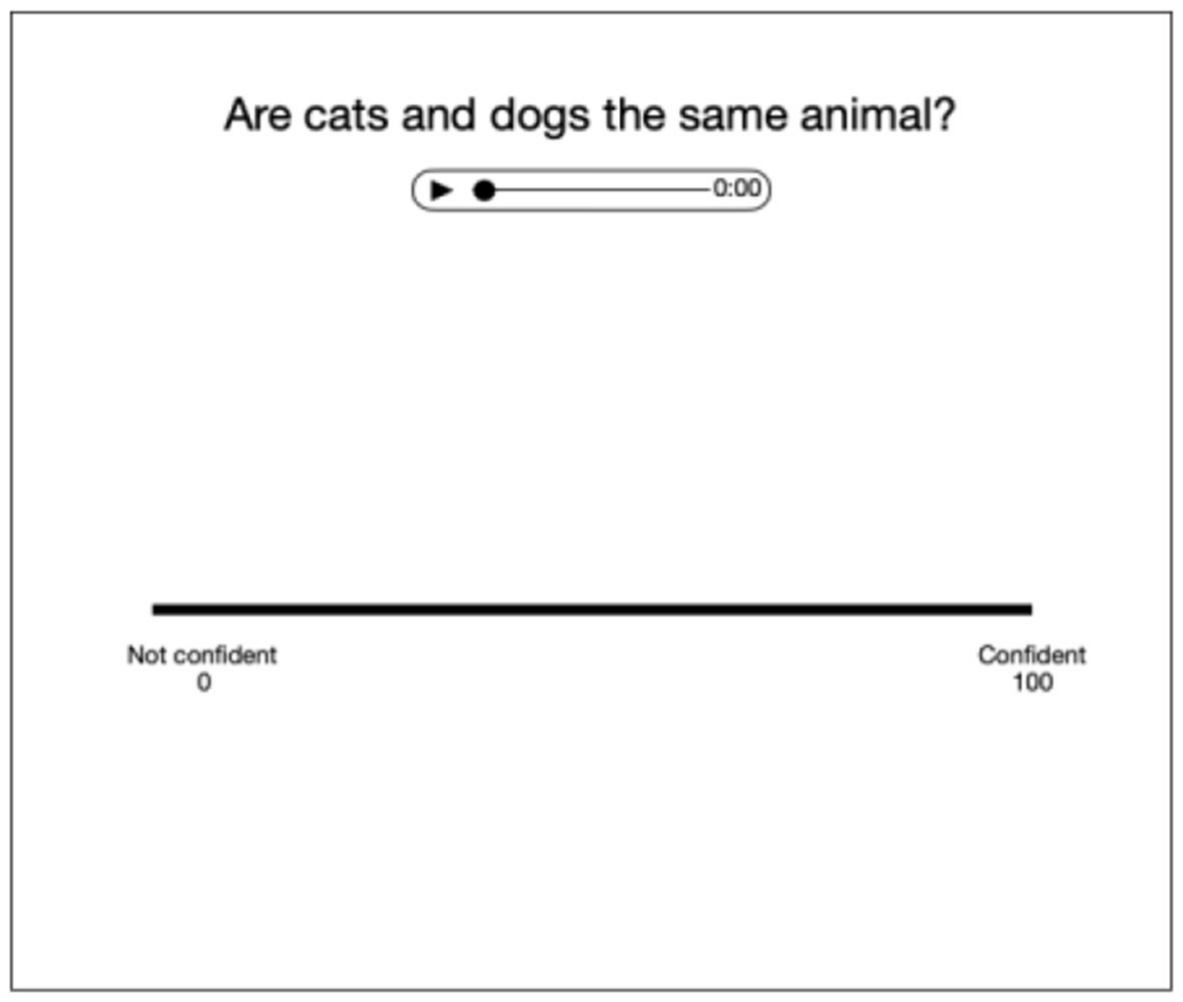
Sample display screen associated with the rating scale for the social judgments of confidence.

After informed consent was obtained, listeners were randomly assigned to the single or multi-talker (mixed or blocked) conditions. On each experimental trial (single = 24 trials; mixed/blocked = 96 trials), participants were presented with an orthographic presentation of one of the three questions (see Table 1 for questions) and were asked to rate on a scale from 0 to 100, how confident the talker sounded in their response to the question (see Figure 2). All sound files and question pairings were randomly presented to participants based on the between-subjects condition they were assigned.

## 4 Results

Linear mixed random effects models were used to evaluate ratings of confidence as a function of the talker's gender (female voice; male-pitched voice) and intonation contour (declining intonation; rising intonation). Maximal random effect structures with subject and questions^2^ set as random intercepts were implemented based on model convergence; simpler models were chosen when model convergence was an issue using the backward removal of random slopes until convergence was met. Three separate analyses were conducted across the three experimental conditions (single, multiple talkers mixed, multiple talkers blocked) because of the different lengths and structures of the trials. All models were evaluated in R (R Core Team, 2023) using lme4 (Bates et al., 2015), with emmeans (Lenth and Lenth, 2018) and MuMIn (r.squaredGLMM; Barton and Barton, 2015) used to report contrasts and effect sizes, respectively. All stimuli, analyses, and data are available on Open Science Framework (https://osf.io/y63rp/).

### 4.1 Single talker condition

**Figure 3:**
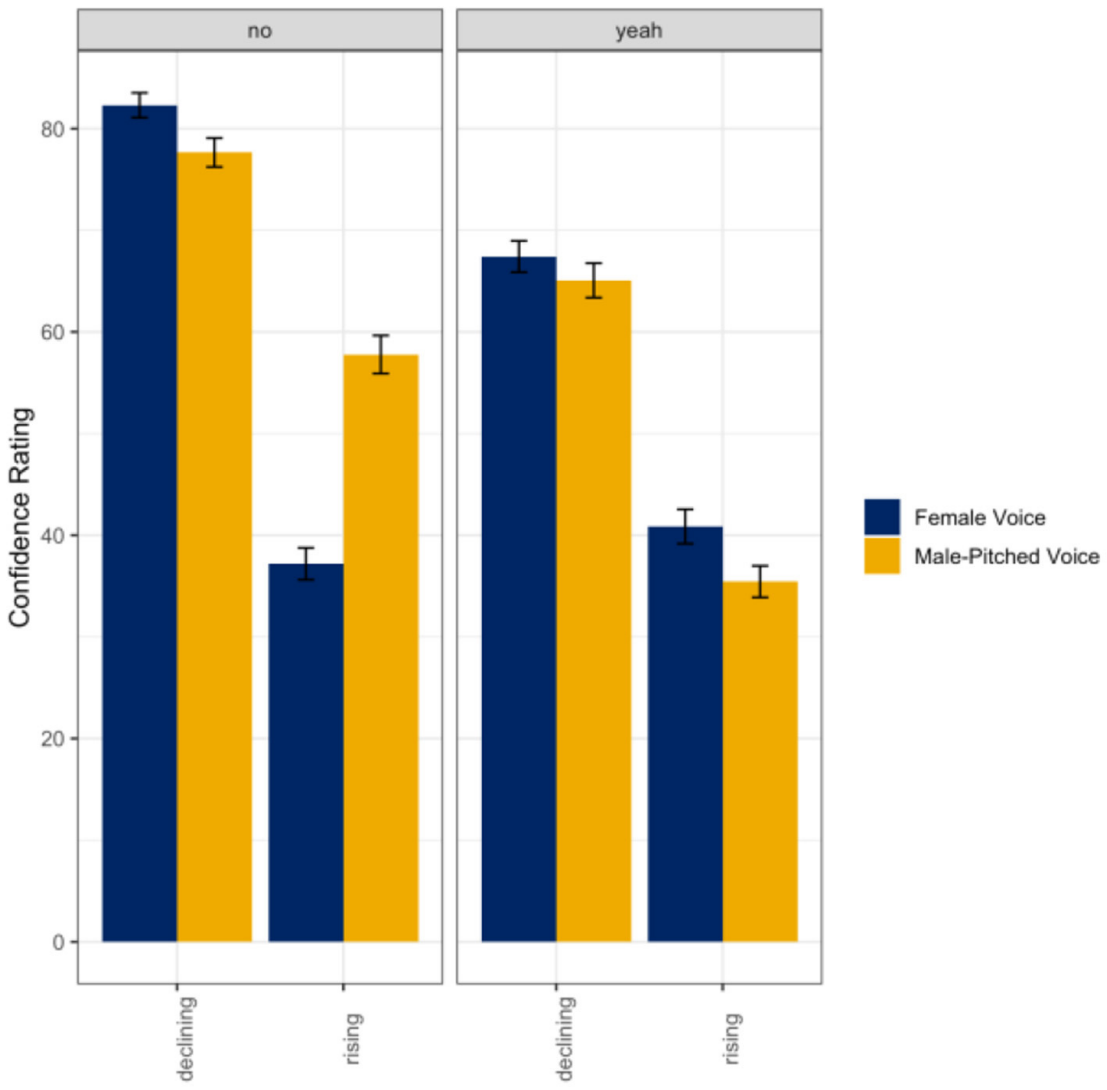
Means and standard errors for listener ratings of confidence as a function of the talker's gender and intended intonation contour meaning (declining intonation = confident; rising intonation contour = not confident) for the word *no* **(left panel)** and the word *yeah* **(right panel)** in the single talker condition.

A linear mixed random effects model was used to evaluate listener ratings of confidence as a function of the talker's gender (female voice; male-pitched voice), intonation (declining; rising), and response word (*yeah*; *no*) in the single talker condition. Results indicated a main effect of the talker's gender (ß = −4.65, *SE* = 1.92, *t* = −2.42, *p* = 0.02), intonation (ß = −45.11, *SE* = 2.97, *t* = −15.22, *p* < 0.001), and response word (ß = −14.88, *SE* = 2.00, *t* = −7.43, *p* < 0.001). There were also significant interactions between the talker's gender and intonation (ß = 25.24, *SE* = 2.48, *t* = 10.17, *p* < 0.001), intonation and response word (ß = 18.55, *SE* = 2.48, *t* = 7.47, *p* < 0.001), and the talker's gender, intonation, and response word (ß = −28.32, *SE* = 3.51, *t* = −8.07, *p* < 0.001) with all fixed effects accounting for ~57% (*R*^2^) of the variance in listener ratings of confidence. Overall, the male-pitched voices were rated as significantly more confident than the female voices, rising intonations were rated as significantly less confident than the declining intonations, and *no* utterances were rated as more confident than *yeah* utterances. As seen in Figure 3, listeners only appeared to differentiate the word *no* spoken with a rising intonation when uttered by a female voice such that the female voice using this intonation pattern was perceived to be significantly less confident than the male-pitched voice (*p* < 0.001). However, listeners did not significantly differentiate between gender in the declining intonation (*no*: *p* = 0.23; *yeah*: *p* = 0.93) or rising intonation (*yeah*: *p* = 0.09).

### 4.2 Multiple talkers mixed condition

**Figure 4:**
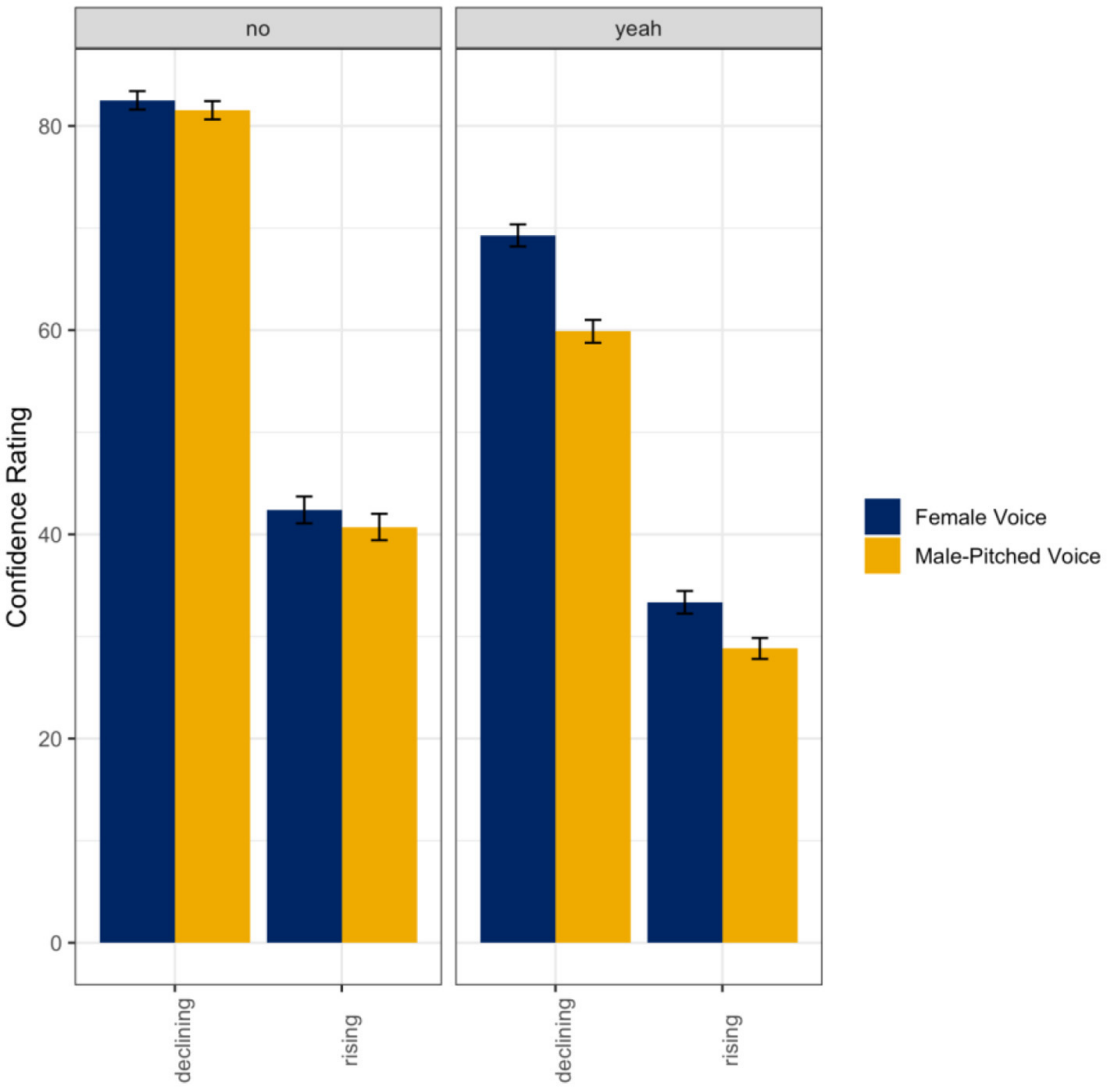
Means and standard errors for listener ratings of confidence as a function of the talker's gender and intended intonation contour meaning (declining intonation = confident; rising intonation contour = not confident) for the word *no* **(left panel)** and the word *yeah* **(right panel)** in the multiple talkers mixed condition.

A linear mixed random effects model was used to evaluate listener ratings of confidence as a function of the talker's gender (female voice; male-pitched voice), intonation (declining; rising), and response word (*yeah*; *no*) in the multiple talkers mixed condition. Results indicated a main effect of intonation (ß = −40.11, *SE* = 2.97, *t* = −13.50, *p* < 0.001) and response word (ß = −13.23, *SE* = 2.06, *t* = −6.41, *p* < 0.001). Additionally, there was also a significant interaction between the talker's gender and response word (ß = −8.43, *SE* = 1.87, *t* = −4.52, *p* < 0.001), intonation and response word (ß = 4.18, *SE* = 1.87, *t* = 2.24, *p* = 0.03), and talker's gender, intonation, and response word (ß = 5.59, *SE* = 2.64, *t* = 2.11, *p* = 0.03) with all fixed effects entered into the model accounting for ~51% (*R*^2^) of the variance in listener ratings of speaker confidence. Overall, the women talkers were rated as more confident, the rising intonation was rated as less confident than the declining intonation, and the word *no* recruited higher ratings of confidence. As seen in Figure 4, listeners rated the women's voices as more confident when they said *yeah* compared to the male-pitched voice (*p* < 0.001). However, listeners did not differentiate between declining intonation (*no*) or rising intonation (*no*: *p* = 0.95; *yeah*: *p* = 0.053). It should be noted that there was a marginal increase (though not significant) in women using rising intonation when saying *yeah*.

### 4.3 Multiple talkers blocked condition

**Figure 5:**
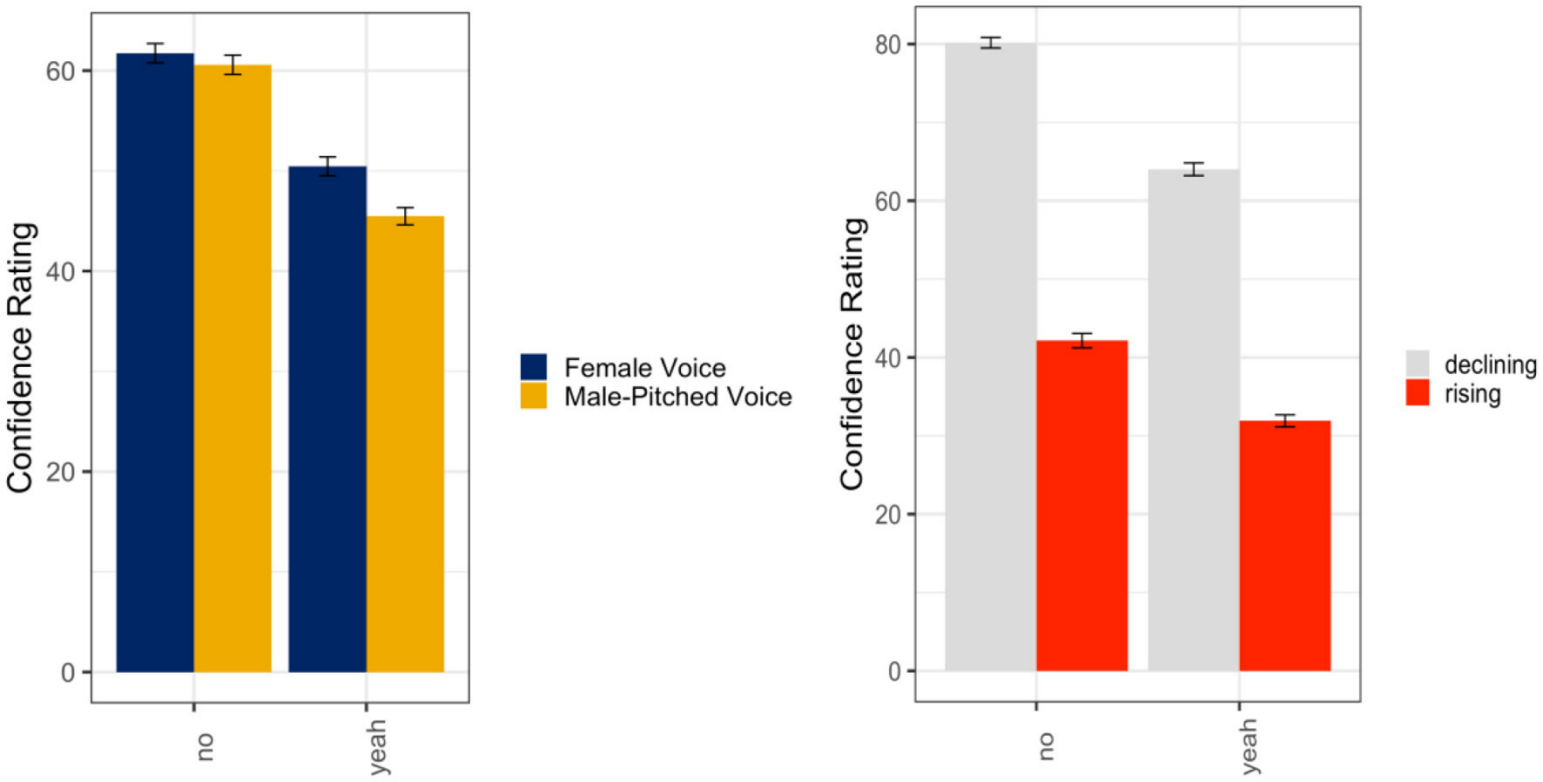
Means and standard errors for listener ratings of confidence as a function of the word and talker's gender **(left panel)** and intonation (declining intonation = confident; rising intonation contour = not confident) **(right panel)** in the multiple talkers blocked condition.

A linear mixed random effects model was used to evaluate listener ratings of confidence as a function of talker order (female voice first; male-pitched voice first), talker's gender (female voice; male-pitched voice), intonation (declining; rising), and response word (*yeah*; *no*) in the multiple talkers blocked condition. The results indicated a main effect of intonation (ß = −38.33, *SE* = 4.34, *t* = −8.95, *p* < 0.001) and response word (ß = −17.05, *SE* = 1.98, *t* = −8.57, *p* < 0.001). There was a significant interaction between the talker's gender and the response word (ß = −5.38, *SE* = 2.81, *t* = −1.91, *p* = 0.06) and a significant interaction between intonation and response word (ß = 9.35, *SE* = 2.81, *t* = 3.23, *p* = 0.001) with all fixed effects entered into the model accounting for ~46% (*R*^2^) of the variance in listener ratings of confidence. Again, listeners rated utterances with rising intonation and *yeah* utterances as less confident. As seen in Figure 5 (left panel), listeners' ratings of confidence for the female voices trended toward more confidence when they used the rising intonation relative to the male-pitched voices, but rising intonation also received lower ratings of confidence for both response words (p < 0.001), but more so for the *yeah* utterances (*p* < 0.001; Figure 5, right panel).

## 5 Discussion

Stereotypes have the potential to shape how listeners view the world; however, statistical properties of the environment (i.e., what is being said, who is saying it, and how it is said) may modify how listeners interpret language. This led us to ask the following research question: How did context drive listeners' social judgments of confidence? It was hypothesized that both vocal gender cues and linguistic cues would impact social judgments of confidence. Overall, it was hypothesized that women and woman-typed language (i.e., affirmation) would be rated less confidently than the male-pitched voice in the single talker condition, but this effect would disappear when many women and male-pitched speakers produced the same cue to confidence. Results, in fact, indicated that language and socio-indexical cues to gender and vocal affective expression impacted social judgments of confidence in different ways depending on the listening context. Across all three tasks, *yeah* recruited lower ratings of confidence relative to *no* utterances (consistent with study hypotheses). However, when the female voices said *yeah* in the mixed and blocked conditions, this increased ratings of confidence. Mulac et al. (2013) explain that social schemas commonly develop about how men and women *should* normatively communicate—i.e., *gender-linked language effect—*and because women are expected to be agreeable (e.g., Prentice and Carranza, 2002; Judge et al., 2012), when they produced an affirmation, this may have boosted listeners social judgments of confidence (e.g., potentially due to linguistic expectancy bias; Maass, 1999; Wigboldus et al., 2000; Cadinu et al., 2003). However, when *no* was uttered, we saw higher ratings of confidence for both female and male-pitched voices across all three tasks. Even though recent studies have shown that men tend to produce more negation (e.g., Newman et al., 2008), some argue that negation language is *female*-typed (Mulac et al., 1986), but this effect seems to be largely dependent on context (e.g., who the speaker is conversing with; Hancock et al., 2015). It could be that negation is not treated in a similar way as affirmation, as it may not hold the same stereotypical characteristics as affirmation. However, intonation differences impacted ratings of confidence in predicted ways.

As expected and consistent with the study hypotheses, rising intonation garnered lower ratings of confidence than the declining intonation contour. This finding is consistent with other naturalistic studies that report similar acoustic intonation patterns (i.e., declining and rising intonation) associated with vocal confidence and lack of confidence (Roche et al., 2019, 2022). Interestingly, however, listeners rated the confidence of the speakers based on perceived gender differently in the multiple talkers and single talker tasks such that female voices were rated as less confident overall when they said *no* with a rising intonation, relative to male-pitched voices in the single talker task. This effect washed out in the multiple talkers condition even though the same female and male-pitched voices of the single talker task were included in the multi-talker tasks.

Consistent with findings from other speech perception studies, listeners may have used stereotyped predictors to track the statistical distributions about the talkers as a means to make speech recognition simpler (Kraljic and Samuel, 2006, 2007; Clayards et al., 2008; McMurray and Jongman, 2011). However, in the multi-talker conditions, listeners may have learned that the rising intonational cue was equally distributed across all talkers. This may have prompted them to realize that there was no communicative benefit that warranted the tracking of separate cue distributions for the different talker groups (i.e., gender). This suggests that listeners in the current study seemed to be *ideal adapters* (Kleinschmidt, 2019), as they exhibited expected trends away from stereotyped responses in the multiple talkers conditions.

Moreover, the tracking of conditional distributions and statistical regularity of cues in the communicative environment has interesting implications for cognition and social decision-making. Considering dual process models of information processing, a triggering cue plays a very important role in prompting the processing path to aid interpretation (e.g., Chaiken, 1980; Bohner et al., 1995). When processing speech and socio-indexical cues, the decision to rely on heuristic vs. systematic-based processing is dependent on the available cues that trigger processing along one path or the other. Heuristics can vary the strength that is needed to trigger when expectancies vary (e.g., weak cues are needed for strong expectancies; Bruner, 1957; Bohner et al., 1995, p. 37). In the single-talker condition, the rising intonation cue, which has been shown to be predominantly a woman-typed cue (Roche et al., 2019, 2022), may have been strong enough to trigger a stereotype because of the strong expectation that women are prescriptively agreeable and less confident than men (Prentice and Carranza, 2002).

However, in the multiple talkers conditions (mixed and blocked), because many female and male-pitched voices were producing this cue, (1) intonation may have been less salient to trigger the prescriptive stereotype about women and confidence or (2) the existence of more than one cue systematically being present in the listening context could have dampened or weakened the trigger, altering the gender stereotype expectation. It is possible that the cue may have been perceived less saliently as a gender cue (i.e., a weaker trigger of the heuristic) because the expectation was altered due to the distributional statistics of the cue used across both genders of speakers (i.e., Kraljic and Samuel, 2006, 2007; Clayards et al., 2008; McMurray and Jongman, 2011; Kleinschmidt, 2019). Therefore, the listeners adapted or adjusted their social judgments based on the statistical properties of the listening context. In other words, because both women and men are known to produce the rising intonation cue in a lack of confidence context (Jiang and Pell, 2015; Roche et al., 2019, 2022), the sheer number of people producing this cue reduced its weightage, which avoided the triggering of the heuristic, stereotypical response that women are less confident than men. In fact, when we visually inspected confidence ratings for the female and male-pitched voices over time in the mixed condition, there is a clear visual pattern that supports this assumption. For instance, the male-pitched voices were rated more confidently at the beginning of the task across all intonation conditions, but this slightly decreased over the course of the experiment. An opposite pattern was apparent for the women talkers—their rates were lower at the beginning of the task but gradually increased toward the end of the experiment (see OSF Supplementary Figure 1).

Though the findings from the current study provide interesting insights into how the cognitive system parses and handles communicative cues, this study is not without limitations. As with many speech perception and social categorization tasks, ecological validity and mundane realism are of concern as participants may not make social judgments about vocal confidence in a similar way in real-world contexts. Nevertheless, the findings from this study do replicate findings from two other studies investigating vocal cues to confidence in that women tend to be rated as less confident than men (Roche et al., 2019, 2022), providing strength to the results of the present study. Another major limitation of the current study is that only college-aged adults who were primarily women were recruited as a convenience sample, which may further impact the generalizability of the results as college-aged women may view gender and gender roles in different ways than the general public, men, and other gender identities. This study should be conducted again to determine if the results are generalizable or specific to this convenient sample. The words chosen for affirmation and negation categories also produced different effects. It could be that the use of *yeah* and *no* behaved very differently compared to other words of negation and affirmation, and the use of *yeah* (i.e., the slang version of yes) may have also impacted social judgments in other pragmatic ways not accounted for in the current study. Future studies should consider how different lexical choices may impact social judgments of confidence (e.g., saying *true* or *false*). Lastly, the current study only manipulated vocal effects from women talkers. This was mostly done because the acoustic manipulation attempted for a male voice to a female-pitch voice produced a gender category that sounded neither like a biological male nor a biological female. Therefore, future studies should evaluate how gender along a continuum and not a binary contributes to our understanding of social judgments and stereotypes about gender and confidence.

To conclude, though many listeners held common stereotypes about the gender of the talker(s) and how it related to social judgments of confidence, statistical properties of the environment appeared to matter. This is important to learn, as we may be able to dispel common and harmful stereotypes about women, especially in professional spaces, that may disrupt their ability to progress in their professional careers. The findings from the current study show that these communicative cues, even when not consistent with gender stereotypes, may become less salient when many people use the cues. Both women and men are known to use rising intonation as a cue to indicate a lack of confidence, among other communicative contexts, but when many men and women produce the same cue, it loses its gender-stereotyped effect. Consider how the general public may judge political candidates based on gender. Cassese and Holman (2018) found that both female and male candidates are vulnerable to negative stereotypes, but these types of threats were much more severe for women with democratic leanings. It could be that in these types of political contexts, when one woman and a man debate, listeners may be more inclined to make stereotyped social judgments. However, if there is a panel of women and men, results from this study suggest that listeners may abandon stereotyped heuristics and could more equitably judge the confidence of the panelists. Therefore, these findings have important implications for how women communicate in public spheres. It is important to replicate this study in other communicative contexts, such as political debates using direct vs. indirect language, etc. This will help determine if the principles discussed still hold constant and whether women professionals in public settings need to adapt their communication style to move away from harmful stereotypes and heuristics.

## Data availability statement

The datasets presented in this study can be found in online repositories. The names of the repository/repositories and accession number(s) can be found in the article/supplementary material.

## Ethics statement

The studies involving humans were approved by Kent State University Institutional Review Board. The studies were conducted in accordance with the local legislation and institutional requirements. The participants provided their written informed consent to participate in this study.

## Author contributions

All authors have contributed to the development of the submitted manuscript, have reviewed the document, and agree to submit it in its current form.

## Footnotes

^1^Typically, a linear mixed random effects model is preferred for these types of analysis. However, there were convergence and fit issues with the linear model and an ANOVA was chosen instead.

^2^The questions were evaluated as a fixed effect in a larger model but produced no significant effects for either experimental condition. Therefore, they were set as a random intercept in the single model but dropped as a random intercept in the multiple talkers condition due to convergence issues.
